# The significance of oral streptococci in patients with pneumonia with risk factors for aspiration: the bacterial floral analysis of 16S ribosomal RNA gene using bronchoalveolar lavage fluid

**DOI:** 10.1186/s12890-016-0235-z

**Published:** 2016-05-11

**Authors:** Kentaro Akata, Kazuhiro Yatera, Kei Yamasaki, Toshinori Kawanami, Keisuke Naito, Shingo Noguchi, Kazumasa Fukuda, Hiroshi Ishimoto, Hatsumi Taniguchi, Hiroshi Mukae

**Affiliations:** Department of Respiratory Medicine, School of Medicine, University of Occupational and Environmental Health, Japan, 1-1 Iseigaoka, Yahatanishiku, Kitakyushu city, Fukuoka 807-8555 Japan; Department of Microbiology, University of Occupational and Environmental Health, Japan, 1-1 Iseigaoka, Yahatanishiku, Kitakyushu city, Fukuoka 807-8555 Japan; Second Department of Internal Medicine, Nagasaki University School of Medicine, 1-7-1 Sakamoto, Nagasaki, 852-8501 Japan

**Keywords:** Aspiration pneumonia, Aspiration risks, Oral streptococci, Anaerobes, Bacterial floral analysis; 16S ribosomal RNA gene

## Abstract

**Background:**

Aspiration pneumonia has been a growing interest in an aging population. Anaerobes are important pathogens, however, the etiology of aspiration pneumonia is not fully understood. In addition, the relationship between the patient clinical characteristics and the causative pathogens in pneumonia patients with aspiration risk factors are unclear. To evaluate the relationship between the patient clinical characteristics with risk factors for aspiration and bacterial flora in bronchoalveolar lavage fluid (BALF) in pneumonia patients, the bacterial floral analysis of 16S ribosomal RNA gene was applied in addition to cultivation methods in BALF samples.

**Methods:**

From April 2010 to February 2014, BALF samples were obtained from the affected lesions of pneumonia via bronchoscopy, and were evaluated by the bacterial floral analysis of 16S rRNA gene in addition to cultivation methods in patients with community-acquired pneumonia (CAP) and healthcare-associated pneumonia (HCAP). Factors associated with aspiration risks in these patients were analyzed.

**Results:**

A total of 177 (CAP 83, HCAP 94) patients were enrolled. According to the results of the bacterial floral analysis, detection rate of oral streptococci as the most detected bacterial phylotypes in BALF was significantly higher in patients with aspiration risks (31.0 %) than in patients without aspiration risks (14.7 %) (*P* = 0.009). In addition, the percentages of oral streptococci in each BALF sample were significantly higher in patients with aspiration risks (26.6 ± 32.0 %) than in patients without aspiration risks (13.8 ± 25.3 %) (*P* = 0.002). A multiple linear regression analysis showed that an Eastern Cooperative Oncology Group (ECOG) performance status (PS) of ≥3, the presence of comorbidities, and a history of pneumonia within a previous year were significantly associated with a detection of oral streptococci in BALF.

**Conclusions:**

The bacterial floral analysis of 16S rRNA gene revealed that oral streptococci were mostly detected as the most detected bacterial phylotypes in BALF samples in CAP and HCAP patients with aspiration risks, especially in those with a poor ECOG-PS or a history of pneumonia.

## Background

Pneumonia is the third and ninth most common cause of death in Japan [1] and the United States [2], respectively. As the population ages, the mortality associated with pneumonia is increasing, especially among the elderly population of over 65 years of age [1].

The detection of the causative pathogens in pneumonia patients is generally based on sputum culture [3], but molecular methods using 16S ribosomal RNA gene sequence have yielded new bacteriological information for pneumonia [4–7]; specifically, that anaerobes and oral streptococci are important pathogens for community-acquired pneumonia (CAP) [5], healthcare-associated pneumonia (HCAP) [7], ventilator-associated pneumonia (VAP) [4, 6], and bacterial pleurisy [8]. The bacterial floral analysis of 16S rRNA gene we used have the advantages that can evaluate the ratio of each bacterial phylotype in each sample in addition to the ability to detect bacterial phylotypes that are difficult or impossible to be cultured [5, 7, 8].

Aspiration risks are important factors in elderly patients with pneumonia [9], as it can be highly influential to the causative pathogens. The guidelines for pneumonia recommend the use of effective antimicrobials to anaerobes in patients who are at risk for aspiration [10], however, there is insufficient etiological information of causative bacteria in these patients [11].

The objective of this study was to evaluate detected bacterial phylotypes by the bacterial floral analysis of 16S rRNA gene in addition to cultivation methods in bronchoalveolar lavage fluid (BALF) in patients with CAP and HCAP, and also to assess the relationship between the patient clinical characteristics with risk factors for aspiration and the most detected bacterial phylotypes by the bacterial floral analysis, using both simple and multiple regression analyses.

## Methods

### Study population

**Fig. 1:**
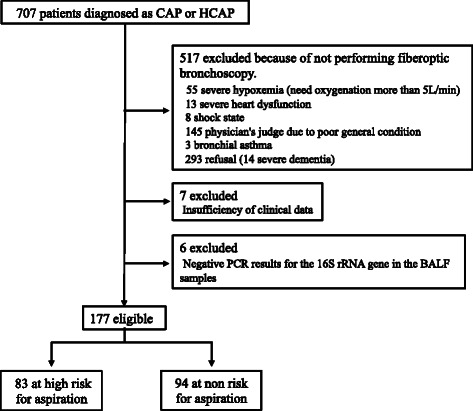
A flow chart of the participants in this study. Definitions of abbreviations: CAP, community-acquired pneumonia; HCAP, healthcare-associated pneumonia; BALF, bronchoalveolar lavage fluid

We recruited CAP (83) and HCAP (94) patients at the University of Occupational and Environmental Health, Japan and referred community hospitals between April 2010 and February 2014. Among these patients, 61 CAP [5] and 78 HCAP [7] patients were included in previous studies, and three of the CAP patients and four of the HCAP patients in the past studies were excluded because of insufficient samples or data (Fig. 1). The patients were classified into two groups: pneumonia patients with aspiration risk factors and those without. This study was approved by the Human and Animal Ethics Review Committee of the University of Occupational and Environmental Health, Japan (No.09–118). All patients provided written informed consent.

### Definitions

CAP and HCAP were defined according to the Infectious Diseases Society of America/American Thoracic Society guidelines [3]. The criteria for aspiration risk factors defined by Marik et al. [12] were used, that consist of having neurologic dysphagia, disruption of the gastroesophageal junction, or anatomical abnormalities of the upper aerodigestive tract. Patients with gastroesophageal disorders were categorized as “disruption of the gastroesophageal junction”. Eastern Cooperative Oncology Group (ECOG) performance statuses (PS) were used, Grade 0; fully active, able to carry on all pre-disease performance without restriction, Grade one; restricted in physically strenuous activity but ambulatory and able to carry out work of a light or sedentary nature, e.g., light house work, office work, Grade two; ambulatory and capable of all self-care but unable to carry out any work activities, up and about more than 50 % of waking hours, Grade three; capable of only limited self-care, confined to bed or chair more than 50 % of waking hours, Grade four; Completely disabled, cannot carry on any self-care, totally confined to bed or chair, grade five; dead [13]. In this study, *Streptococcus mutans* group and the *S. mitis* group, the *S. salivarius* group, and the *S. anginosus* group were included as “oral streptococci” except for *S. pneumoniae*.

### Data and sample collection

The clinical information of the patients, including the laboratory and radiological information on chest X-rays and computed tomography (CT) findings were collected. BALF specimens using 40 ml of sterile saline were obtained from pneumonia lesions, as described previously [5, 7].

### Total cell count and cell lysis efficiency analysis

The total bacterial cell count and cell lysis efficiency were evaluated using epifluorescent microscopy, as previously described [5, 7, 8].

### Microbiological evaluation using cultivation methods

**Fig. 2:**
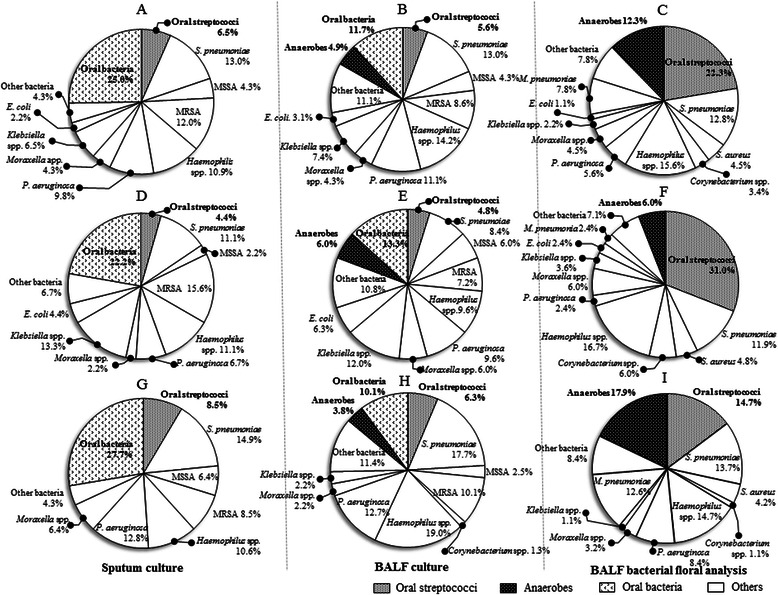
The percentages of the detected bacteria by sputum cultivation, and cultivation and the bacterial floral analysis of 16S ribosomal RNA gene in bronchoalveolar lavage fluid. The percentages of the bacterial species detected by the cultivation using sputum samples (**a**, **d** and **g**) or bronchoalveolar lavage fluid (BALF) (**b**, **e** and **h**), and the most detected bacterial phylotypes detected by the bacterial floral analysis of 16S rRNA gene in BALF (**c**, **f** and **i**). The numbers in the Figures describe the percentages of detected bacteria in patients of all (**a**, **b** and **c**), with aspiration risks (**d**, **e** and **f**) and without aspiration risks (**g**, **h** and **i**), respectively. The denominators in **a**, **b**, **d**, **e**, **g** and **h** are the numbers of patients in whom some bacterial species were cultured, and the bacterial floral analysis of 16S rRNA gene detected at least one or more bacterial phylotypes in all of the BALF samples (**c**, **f** and **i**). Ninety-one patients could not produce any sputum for examination at hospital admission (**a**, **d** and **g**). All members of the *Streptococcus mutans* and *S. mitis* groups, the *S. salivarius* group, and the *S. anginosus* group were included as “oral streptococci” except for *S. pneumoniae* (**c**, **f** and **i**). Definitions of abbreviations: MSSA, methicillin-susceptible *Staphylococcus aureus*; MRSA, methicillin-resistant *S. aureus*

The cultivation of BALF and sputum samples was performed as previously described [5, 7]. Positive results were recorded for all bacterial species. The presence of more than one positive BALF and/or sputum culture is described in the “Total” column of Table 2, while the positive culture results for BALF and sputum samples are presented in the corresponding columns of Fig. 2.

### The bacterial floral analysis of 16S ribosomal RNA gene

The DNA of 16S rRNA genes in BALF samples was extracted and the partial 16S rRNA gene fragments (approximately 580 base pairs) were amplified by a polymerase chain reaction (PCR) method with universal primers (341F and 907R). The amplified products were cloned into *Escherichia coli* using a TOPO TA cloning kit (Invitrogen, Carlsbad, CA), and the nucleotide sequences of 96 randomly chosen clones were determined, and comparison of the detected sequences with the type strains using the basic local alignment search tool (BLAST) algorithm were performed, as described previously [5, 7, 8]. Using this method, each bacterial phylotype was precisely estimated including differentiation of *S. pneumoniae* and other streptococci in the BALF.

### Statistical analysis

The SPSS software package (version 19) was used, and Fisher’s exact test for tables (2 × 2), and the Mann–Whitney *U* test were applied. *P* < 0.05 was considered significant. When the most detected bacterial phylotypes were considered as dependent variables and clinical variables were considered as independent variables, a simple regression analysis was used. In addition, a multiple linear regression analysis was also performed. The variables were included in the original simple regression analysis model if their univariable *P* values were less than 0.05.

## Results

### Patient characteristics

The characteristics of 177 patients (CAP 83, HCAP 94) are shown in Table 1, and 46.9 % (83/177) patients had aspiration risks. Compared to the patients without aspiration risks, the patients with aspiration risks were significantly older, including more males and HCAP patients, higher rates of patients who resided in a nursing home or an extended care facility, lower average body mass indices (BMI), higher percentages of patients with median ECOG-PS of 3–4 of premorbid conditions, patients using antipsychotic drugs and patients with history of pneumonia within the previous 1 year (Table 1). The patients with aspiration risks demonstrated significantly higher percentages of patients with orientation disturbance, lower hematocrit values and average serum albumin levels, and more patients with posterior dominant distribution of infiltration on chest CT and more patients with a Pneumonia Severity Index (PSI) score of VI-V than the patients without aspiration risks (Table 1).

**Table 1:** Characteristics of patients with or without risk factors for aspiration

Variables	Presence of risk	Absence of risk	*p* value
	factors for aspiration	factors for aspiration	
	(*n*=83)	(*n*=94)	
Age, years, mean±SD	76.6 (9.5)	64.5 (19.4)	<0.001
Gender, male; *n* (%)	58 (69.9)	48 (51.1)	0.011
HCAP; *n* (%)	59 (71.1)	35 (37.2)	<0.001
Hospitalization for >2 days in the preceding 90 days	34 (41.0)	27 (28.7)	0.087
Residence in a nursing home or extended care facility	29 (34.9)	5 (5.3)	<0.001
Home infusion therapy (including antibiotics)	10 (12.0)	8 (8.5)	0.437
Chronic dialysis during the preceding 30 days	0 (0.0)	1 (1.1)	0.346
Home wound care	0 (0.0)	0 (0.0)	
BMI, mean±SD^a^	19.6 (4.8)	20.7 (4.5)	0.038
ECOG PS, median (IQR)	2 (0.5–3.5)	1 (0.5–1.5)	<0.001
0–1; *n* (%)	28 (33.7)	73 (77.7)	<0.001
2; *n* (%)	22 (26.5)	12 (12.8)	
3–4; *n* (%)	33 (39.8)	9 (9.6)	<0.001
Smoking; *n*, (%)^b^			
Smoker	7 (10.0)	9 (10.7)	
Ex-smoker	30 (42.9)	30 (35.7)	
Non smoker	33 (47.1)	45 (53.6)	
B.I^c^	493.7 (606.1)	449.4 (641.7)	0.454
Comorbidity; *n*, (%)			
Chronic respiratory disease	22 (26.5)	38 (40.4)	0.051
COPD	11 (13.3)	23 (24.5)	0.059
Bronchiectasis	6 (7.2)	12 (12.8)	0.224
Lung cancer	7 (8.4)	3 (3.2)	0.132
Interstitial pneumonia	6 (7.2)	9 (9.6)	0.576
Cerebrovascular disease	30 (36.1)	0 (0.0)	–
Neuromuscular diseases	19 (22.9)	0 (0.0)	–
Dementia	26 (31.3)	0 (0.0)	–
Pharyngeal disorder	7 (8.4)	0 (0.0)	–
Gastroesophageal disorder	27 (32.5)	0 (0.0)	–
Diabetes mellitus	11 (13.3)	22 (23.4)	0.084
Malignancy excluding lung cancer	26 (31.3)	18 (19.1)	0.060
Congestive heart failure	8 (9.6)	8 (8.5)	0.794
Chronic kidney disease	2 (2.4)	5 (5.3)	0.450
Chronic liver disease	6 (7.2)	2 (2.1)	0.103
RA or Sjogren’s syndrome	0 (0.0)	9 (9.6)	0.004
Collagen disease	5 (6.0)	7 (7.4)	0.707
Psychiatric disease	2 (2.4)	4 (4.3)	0.686
Medications; *n* (%)			
Sleeping medications	17 (20.5)	10 (10.6)	0.07
Glucocorticoids (PSL>5 mg/day)	11 (13.3)	7 (7.4)	0.202
Immunosuppressive agent	3 (3.6)	8 (9.5)	0.178
Antipsychotic drugs	7 (8.4)	1 (1.1)	0.027
Antidepressant	3 (3.6)	6 (6.4)	0.504
History of pneumonia within the previous 1 year; *n* (%)	24 (28.9)	15 (16.0)	0.038
Respiratory failure; *n*, (%)	31 (37.3)	30 (31.9)	0.448
Clinical parameters; *n*, (%)			
Orientation disturbance (confusion)	21 (25.3)	8 (8.5)	0.003
Systolic BP<90 mmHg or diastolic BP<60 mmHg	5 (6.0)	2 (2.1)	0.255
Body temperature<35 °C or >40 °C	4 (4.8)	1 (1.1)	0.188
Pulse rate>125 beats/min	5 (6.0)	10 (10.6)	0.271
Laboratory findings			
BUN>10.7 mmol/L	28 (33.7)	24 (25.5)	0.232
Glucose>13.9 mmol/L	3 (3.6)	7 (7.4)	0.339
Hematocrit<30 %	16 (19.3)	6 (6.4)	0.010
Albumin, g/dl mean ± SD^d^	3.05 (0.61)	3.29 (0.57)	0.011
Radiographic findings; *n*, (%)^e^			
Bilateral lung involvement	54 (65.1)	50 (53.8)	0.128
Upper lobe dominant opacity	14 (16.9)	20 (21.5)	0.437
Lower lobe opacity	54 (65.1)	52 (55.9)	0.216
anterior dominant opacity	8 (9.6)	14 (15.1)	0.278
posterior dominant opacity	67 (80.7)	58 (62.4)	0.007
Gravity-dependent opacity	67 (80.7)	67 (72.0)	0.178
Thickening of bronchovascular bundles	37 (44.6)	50 (53.8)	0.224
Pleural effusion	12 (14.5)	19 (20.3)	0.299
PSI score; mean±SD	112.0 (40.3)	81.0 (46.3)	<0.001
I-III	25 (30.1)	57 (60.6)	
VI-V	53 (69.9)	37 (39.4)	<0.001
In hospital mortality	6 (7.2)	6 (6.4)	0.823

Definition of abbreviations: *SD* standard devision, *CAP* community-acquired pneumonia, *HCAP* healthcare-associated pneumonia, *BMI* body mass index, *ECOG PS* eastern cooperative oncology group performance status, *BI* brinkman index, *COPD* chronic obstructive pulmonary disease, *RA* rheumatoid Arthritis, *PSI* pneumonia severity index

^a^BMI, ^b^smoking, ^c^B.I, ^d^serum albumin and, ^e^radiographic findings were evaluated in 142, 154, 153, 168, and 176 patients, respectively

### Total bacterial cell numbers and cell lysis efficiency analysis

The numbers of bacteria in the BALF samples ranged from 1.2 × 10^4^ to 3.7 × 10^9^ (median, 3.7 × 10^9^) cells/mL. The cell lysis efficiency was maintained at ≥90 % in all samples.

### The comparison of conventional cultivation methods and the bacterial floral analysis of 16S ribosomal RNA gene

The results of the conventional cultivation methods and the most detected phylotypes using the bacterial floral analysis of the 16S rRNA gene in the BALF samples are shown in Fig. 2 and Table 2. The bacterial floral analysis of 16S rRNA gene identified one or more bacterial phylotypes in all of the BALF samples, whereas cultivation methods identified some microbes in 81.9 % (145/177) in BALF and 95.3 % (82/86) in sputum samples. The most detected bacterial phylotypes determined in the BALF using the bacterial floral analysis of 16S rRNA gene are shown in Fig. 2 and Table 2. Oral streptococci (22.3 %), *Haemophilus* spp. (15.6 %), *S. pneumoniae* (12.8 %), anaerobes (12.3 %), and *Mycoplasma pneumoniae* (7.8 %) were mostly detected by the bacterial floral analysis of 16S rRNA gene. In contrast, the cultivation methods detected *Haemophilus* spp*.* (12.8 %), *S. pneumoniae* (11.7 %), *Staphylococcus aureus* (11.7 %), and *Pseudomonas aeruginosa* (10.0 %), while oral streptococci and anaerobes were isolated in only 5.0 and 4.4 % of the samples, respectively (Fig. 2 and Table 2).

**Table 2:** Predominant bacteria according to the bacterial floral analysis of 16S rRNA gene and cultivation methods

	Total			Presence of risk factors for aspiration			Absence of risk factors for aspiration		
	Clone Library Method		Culture	Clone Library Method		Culture	Clone Library Method	Culture	
	The predominant phylotypes in BALF	BALF	Sputum	The predominant phylotypes in BALF	BALF	Sputum	The predominant phylotypes in BALF	BALF	Sputum
Gram-positive pathogens									
*Streptococcus pneumoniae*	23 (12.8)	21 (11.7)	12 (13.0)	10 (11.9)	7 (8.0)	5 (12.0)	13 (13.7)	14 (15.2)	7 (13.7)
*Streptococcus* spp.	40 (22.3)	9 (5.0)	6 (6.5)	26 (31.0)	4 (4.5)	2 (4.8)	14 (14.7)	5 (5.4)	4 (7.8)
*Streptococcus anginosus* group	5 (2.8)	1 (0.6)	0 (0.0)	5 (6.0)	1 (1.1)	0 (0.0)	0 (0.0)	0 (0.0)	0 (0.0)
Other *Streptococcus* spp.	35 (19.6)	8 (4.4)	6 (6.5)	21 (25.0)	3 (3.4)	2 (4.8)	14 (14.7)	5 (5.4)	4 (7.8)
*Staphylococcus aureus*	8 (4.5)	21 (11.7)	15 (16.1)	4 (4.8)	11 (12.5)	8 (19.0)	4 (4.2)	10 (10.9)	7 (13.7)
Methicillin-susceptible *S. aureus*	–	7 (3.9)	4 (4.3)	–	5 (5.7)	1 (2.4)	–	2 (2.2)	3 (5.9)
Methicillin-resistant *S. aureus*	–	14 (7.8)	11 (11.8)	–	6 (6.8)	7 (16.7)	–	8 (8.7)	4 (7.8)
*Staphylococcus* spp. (except *S. aureus*)	2 (1.1)	2 (1.1)	0 (0.0)	0 (0.0)	0 (0.0)	0 (0.0)	2 (2.1)	2 (2.2)	0 (0.0)
*Corynebacterium* spp.	6 (3.4)	2 (1.1)	1 (1.1)	5 (6.0)	1 (1.1)	1 (2.4)	1 (1.1)	1 (1.1)	0 (0.0)
*Gemella* spp.	2 (1.1)	0 (0.0)	0 (0.0)	2 (2.4)	0 (0.0)	0 (0.0)	0 (0.0)	0 (0.0)	0 (0.0)
*Lactobacillus* spp.	1 (0.6)	0 (0.0)	0 (0.0)	1 (1.2)	0 (0.0)	0 (0.0)	0 (0.0)	0 (0.0)	0 (0.0)
*Rothia* spp.	1 (0.6)	0 (0.0)	0 (0.0)	0 (0.0)	0 (0.0)	0 (0.0)	1 (1.1)	0 (0.0)	0 (0.0)
Gram-negative pathogens									
*Haemophilus* spp.	28 (15.6)	23 (12.8)	10 (10.8)	14 (16.7)	8 (9.1)	5 (12.0)	14 (14.7)	15 (16.3)	5 (9.8)
*Pseudomonas aeruginosa*	10 (5.6)	18 (10.0)	9 (9.7)	2 (2.4)	8 (9.1)	3 (7.1)	8 (8.4)	10 (10.9)	6 (11.8)
*Pseudomonas* spp. (except *P. aeruginosa*)	0 (0.0)	2 (1.1)	1 (1.1)	0 (0.0)	1 (1.1)	0 (0.0)	0 (0.0)	1 (1.1)	1 (2.0)
*Moraxella* spp.	8 (4.5)	7 (3.9)	4 (4.3)	5 (6.0)	5 (5.7)	1 (2.4)	3 (3.2)	2 (2.2)	3 (5.9)
*Neisseria* spp.	5 (2.8)	0 (0.0)	0 (0.0)	1 (1.2)	0 (0.0)	0 (0.0)	4 (4.2)	0 (0.0)	0 (0.0)
*Klebsiella* spp	4 (2.2)	12 (6.7)	6 (6.5)	3 (3.6)	10 (11.4)	6 (14.3)	1 (1.1)	2 (2.2)	0 (0.0)
*Escherichia coli*	2 (1.1)	5 (2.8)	2 (2.2)	2 (2.4)	5 (5.7)	2 (4.8)	0 (0.0)	0 (0.0)	0 (0.0)
*Enterobacter* spp.	1 (0.6)	2 (1.1)	2 (2.2)	1 (1.2)	2 (2.3)	1 (2.4)	0 (0.0)	0 (0.0)	1 (2.0)
*Acinetobacter* spp.	0 (0.0)	2 (1.1)	0 (0.0)	0 (0.0)	1 (1.1)	0 (0.0)	0 (0.0)	1 (1.1)	0 (0.0)
*Citrobacter* spp.	0 (0.0)	1 (0.6)	1 (1.1)	0 (0.0)	1 (1.1)	1 (2.4)	0 (0.0)	0 (0.0)	0 (0.0)
*Serratia* spp.	0 (0.0)	2 (1.1)	0 (0.0)	0 (0.0)	2 (2.3)	0 (0.0)	0 (0.0)	0 (0.0)	0 (0.0)
*Burkholderia* spp.	0 (0.0)	1 (0.6)	0 (0.0)	0 (0.0)	0 (0.0)	0 (0.0)	0 (0.0)	1 (1.1)	0 (0.0)
*Proteus* spp.	0 (0.0)	1 (0.6)	0 (0.0)	0 (0.0)	1 (1.1)	0 (0.0)	0 (0.0)	0 (0.0)	0 (0.0)
*Pasteurella* spp.	0 (0.0)	1 (0.6)	0 (0.0)	0 (0.0)	0 (0.0)	0 (0.0)	0 (0.0)	1 (1.1)	0 (0.0)
Anaerobic pathogens	22 (12.3)	8 (4.4)	0 (0.0)	5 (6.0)	5 (5.7)	0 (0.0)	17 (17.9)	3 (3.3)	0 (0.0)
*Prevotella* spp.	11 (6.1)	3 (1.7)	0 (0.0)	4 (4.8)	2 (2.3)	0 (0.0)	7 (7.4)	1 (1.1)	0 (0.0)
*Fusobacterium* spp.	5 (2.8)	1 (0.6)	0 (0.0)	1 (1.2)	0 (0.0)	0 (0.0)	4 (4.2)	1 (1.1)	0 (0.0)
*Micromonas* spp.	2 (1.1)	0 (0.0)	0 (0.0)	0 (0.0)	0 (0.0)	0 (0.0)	2 (2.1)	0 (0.0)	0 (0.0)
*Propionibacterium* spp.	2 (1.1)	0 (0.0)	0 (0.0)	0 (0.0)	0 (0.0)	0 (0.0)	2 (2.1)	0 (0.0)	0 (0.0)
*Veillonella* spp.	1 (0.6)	2 (1.1)	0 (0.0)	0 (0.0)	2 (2.3)	0 (0.0)	1 (1.1)	0 (0.0)	0 (0.0)
*Porphyromonas* spp	1 (0.6)	0 (0.0)	0 (0.0)	0 (0.0)	0 (0.0)	0 (0.0)	1 (1.1)	0 (0.0)	0 (0.0)
*Peptostreptococcus* spp.	0 (0.0)	1 (0.6)	0 (0.0)	0 (0.0)	0 (0.0)	0 (0.0)	0 (0.0)	1 (1.1)	0 (0.0)
*Clostridium* spp.	0 (0.0)	1 (0.6)	0 (0.0)	0 (0.0)	1 (1.1)	0 (0.0)	0 (0.0)	0 (0.0)	0 (0.0)
Atypical pathogens									
*Mycoplasma pneumoniae*	14 (7.8)	–	–	2 (2.4)	–	–	12 (12.6)	–	–
*Legionella pneumonia*	1 (0.6)	–	–	1 (1.2)	–	–	0 (0.0)	–	–
*Actinomyces* spp.	0 (0.0)	2 (1.1)	0 (0.0)	0 (0.0)	0 (0.0)	0 (0.0)	0 (0.0)	2 (2.2)	0 (0.0)
*Nocardia* spp.	1 (0.6)	1 (0.6)	0 (0.0)	0 (0.0)	0 (0.0)	0 (0.0)	1 (1.1)	1 (1.1)	0 (0.0)
Oral bacteria	–	37 (20.9)	24 (25.8)	–	16 (18.2)	7 (16.7)	–	21 (22.8)	17 (33.3)
No growth	–	32 (18.1)	4 (4.7)	–	15 (18.1)	1 (2.8)	–	17 (18.1)	3 (6.0)
Not analysed	–	–	91 (51.4)	–	–	47 (56.6)	–	–	44 (46.8)
Total isolates	179^a^	180	93	84^b^	88	42	95^c^	92	51

Data are presented as n (%) unless otherwise stated. Percentages refer to the total number of isolates except “No growth” and “Not analysed”. “No growth” and “Not analysed” refer to the total number of cases. Definition of abbreviations: BALF: bronchoalveolar lavage fluid. ^a^179 isolates of 177 cases (2 cases had two first predominant bacterial species.).^b^84 isolates of 83 cases.^c^95 isolates of 94 cases

### Comparison of the results of the bacterial floral analysis of 16S ribosomal RNA gene between patients with or without aspiration risks

**Fig. 3:**
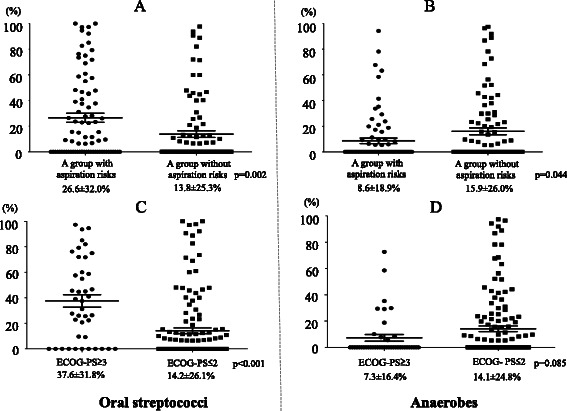
The occupancy of the phylotypes of oral streptococci and anaerobes by the bacterial floral analysis of 16S ribosomal RNA gene according to aspiration risks and ECOG-PS. **a** and **c** represents the occupancy of the phylotypes of oral streptococci, and (**b** and **d**) shows the occupancy of phylotypes of anaerobes, according to aspiration risks (**a** and **b**) and ECOG-PS (**c** and **d**). Using the bacterial floral analysis of 16S rRNA gene in bronchoalveolar lavage fluid, the occupancy rates of oral streptococci were significantly higher in patients with aspiration risks than those in patients without aspiration risks (*P* = 0.02) (**a**), and were also significantly higher in patients with ECOG-PS of ≥3 compared to those in patients with ECOG-PS of ≤2 (*P* < 0.001) (**c**). In contrast, the occupancy rates of anaerobes were significantly higher in patients without aspiration risks than those in patients with aspiration risks (*P* = 0.044) (**b**), and similar trend was observed in patients with ECOG-PS of ≥3 compared to those in patients with ECOG-PS of ≤2 (*P* = 0.085) (**d**). All members of the *Streptococcus mutans* and *S. mitis* groups, the *S. salivarius* group, and the *S. anginosus* group, were included as “oral streptococci” except for *S. pneumoniae*. Abbreviation: ECOG-PS; European Cooperative Oncology Group-Performance Status

**Fig. 4:**
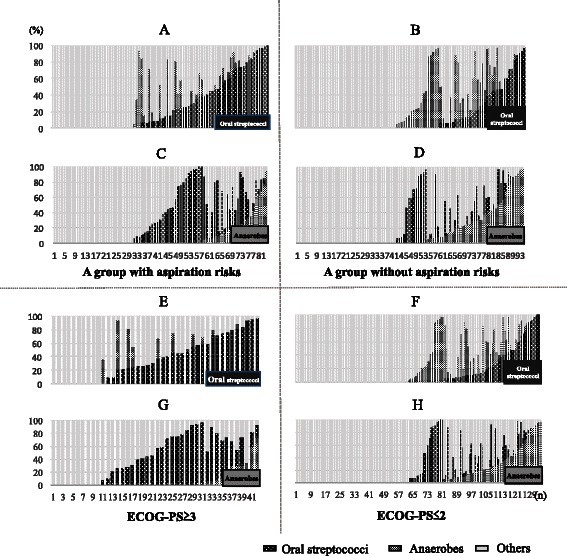
The percentages of oral streptococci (*black*), anaerobes (*gray*) and others (*light gray*) detected by the bacterial floral analysis of 16S ribosomal RNA gene in each bronchoalveolar lavage fluid sample according to risks for aspiration and ECOG-PS. The percentages of oral streptococci (*black*), anaerobes (*gray*) and others (*light gray*) detected by the bacterial floral analysis of 16S rRNA gene in each bronchoalveolar lavage fluid sample in patients with (**a** and **c**) or without (**b** and **d**) risks for aspiration, and those in patients with ECOG-PS of ≥3 (**e** and **g**) or ≤2 (**f** and **h**). **a**, **b**, **e** and **f** focus on the detection rate of oral streptococci (*black*) in each patient, and **c**, **d**, **g** and **h** focus on the detection rate of anaerobes (*gray*) in each patient. All members of the *Streptococcus mutans* and *S. mitis* groups, the *S. salivarius* group, and the *S. anginosus* group were included as “oral streptococci” except for *S. pneumoniae*. Abbreviation: ECOG-PS; european cooperative oncology group performance status

Using the bacterial floral analysis of 16S rRNA gene, oral streptococci as the most detected phylotypes were significantly highly detected in patients with aspiration risks than patients without (31.0 and 14.7 %, respectively, *P* = 0.009); while anaerobes were significantly more detected in patients without aspiration risks (Fig. 2i) than patients with aspiration risks (Fig. 2f) (17.9 and 6.0 %, respectively, *P* = 0.015). Additionally, the percentages of oral streptococci were higher in patients with aspiration risks (Fig. 4a) than those without aspiration risks (Fig. 4b) (Fig. 3a, *P* = 0.002), and anaerobes were significantly more detected in patients without aspiration risks (Fig. 4d) than those with aspiration risks (Fig. 4c) (Fig. 3b, *P* = 0.044).

### Results of simple and multiple linear regression analyses for detecting oral streptococci

Simple regression analysis was performed, using the most detected phylotypes such as oral streptococci (Fig. 2c) as the dependent variable and clinical variables (Table 3) as the independent variable. These variables were included in the original simple regression analysis model if their univariable *P* values were less than 0.05. The significantly correlated factors revealed by a simple regression analysis were; at an age of ≥80, an ECOG-PS of ≥3, residing in a nursing home or long-term care facility, the presence of aspiration risks, and a history of pneumonia within the previous one year (Table 3). The variables were included in the original simple regression analysis model if their univariable P values were less than 0.05. In addition, a multiple linear regression analysis revealed that an ECOG-PS ≥3 and a history of pneumonia were associated with the detection of oral streptococci in the BALF samples by the bacterial floral analysis of 16S ribosomal RNA gene (Table 4).

**Table 3:** Result of simple regresssion analysis for detected oral streptococci

	b	SE (b)	β	*p*-value
Age >80	13.92	4.47	0.229	0.002
BMI<20	−4.07	2.89	−0.106	0.161
Hospitalization for >2 days in the preceding 90 day; *n* (%)	4.15	4.63	0.068	0.371
Residence in a nursing home or long-term care facility	14.48	5.49	0.196	0.009
Home infusion therapy (including antibiotics)	9.88	7.26	0.102	0.170
ECOG PS of premorbid condition>3	23.37	4.87	0.341	<0.001
COPD	−0.28	5.60	−0.004	0.960
Interstitial pneumonia	0.18	7.92	0.002	0.982
Lung cancer	−0.16	9.55	−0.001	0.987
Bronchiectasis	−6.63	7.28	−0.069	0.364
Cerebrovascular disease	7.75	5.85	0.100	0.187
Neuromuscular diseases	8.62	7.09	0.092	0.226
Dementia	8.14	6.20	0.099	0.191
Gastroesophageal disorder	5.72	6.12	0.705	0.351
Pharyngeal disorder	19.48	11.22	0.130	0.084
Presence of the risk factor for aspiration	12.78	4.31	0.219	0.004
Malignancy excluding lung cancer	−1.73	5.10	−0.026	0.735
Psychiatric disease	1.68	12.18	0.010	0.891
Sleeping medications	10.24	6.08	0.126	0.094
Antipsychotic drugs	−4.94	10.61	−0.035	0.642
Antidepressant	10.40	10.01	0.078	0.300
History of pneumonia within the previous 1 year	16.12	4.92	0.241	0.001
Albumin<4 g/dl	−3.25	6.56	−0.037	0.621
Bilateral lung involvement	0.01	4.37	0.0002	0.998
Lower lobe opacity	−4.67	4.40	−0.08	0.291
Posterior dominant opacity	4.56	4.87	0.071	0.351
Gravity-dependent opacity	−7.52	5.27	−0.107	0.155

Definition of abbreviations: *BMI* body mass index, *ECOG PS* eastern cooperative oncology group performance status, *HCAP* healthcare-associated pneumonia, *COPD* chronic obstructive pulmonary disease

**Table 4:** Result of multiple linear regresssion for detected oral streptococci

	b	SE (b)	β	*p*-value
Age>80	6.61	4.62	0.109	0.154
Residence in a nursing home or long-term care facility	−3.28	6.4	−0.044	0.609
ECOG PS of premorbid condition>3	17.52	5.96	0.256	<0.001
Presence of the risk factor for aspiration	5.69	4.54	0.097	0.211
History of pneumonia within the previous 1 year	10.36	4.88	0.155	0.035

b: raw (unstandardised) regression coefficient, *SE* standard error of b coefficient

### Results of the bacterial floral analysis of 16S ribosomal RNA gene in pneumonia patients with an ECOG-PS of ≥3 or ≤2

**Fig. 5:**
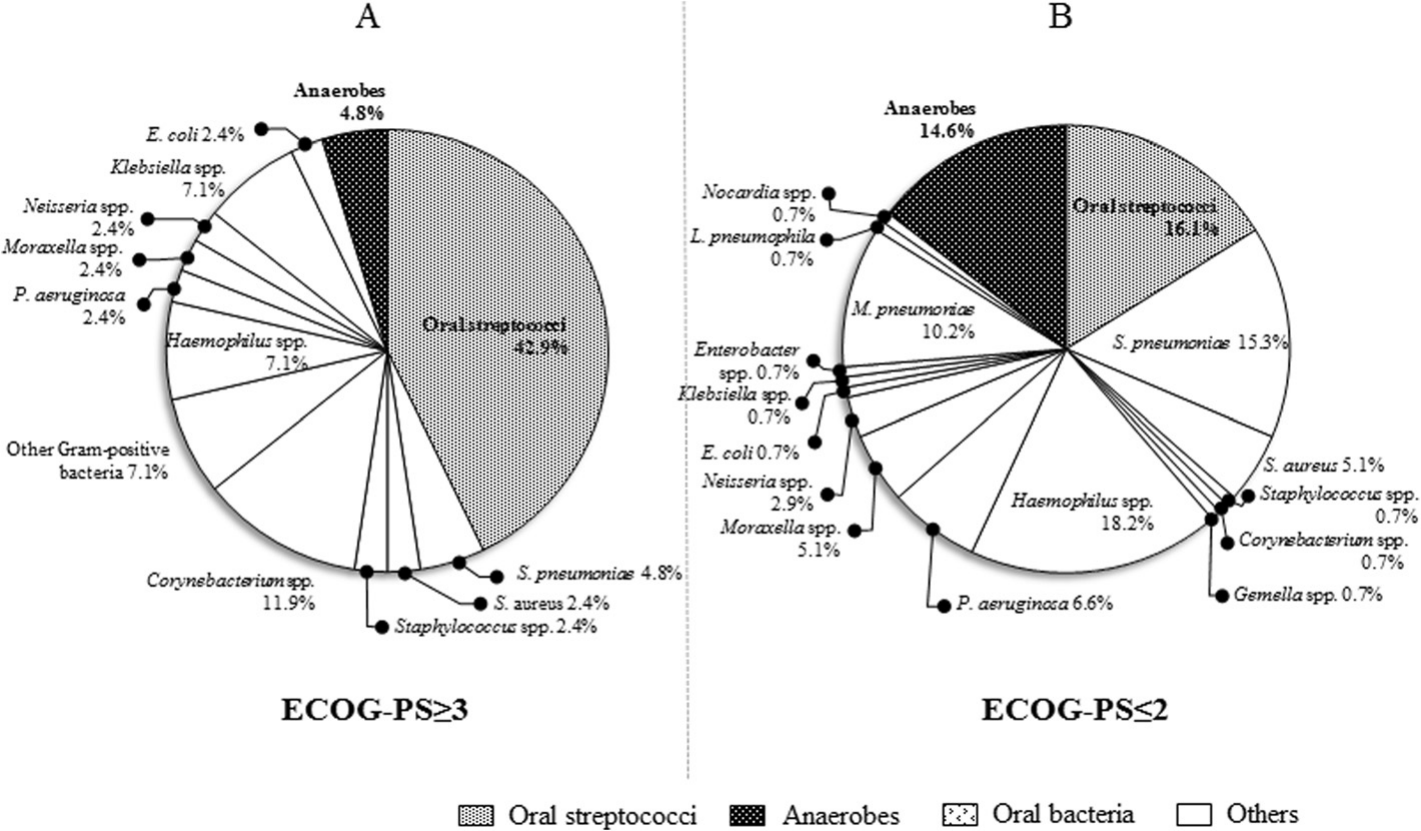
The differences of the percentages of the most detected bacterial phylotypes determined by the bacterial floral analysis of 16S ribosomal RNA gene according to ECOG-PS. The percentages of the most detected bacterial phylotypes by the bacterial floral analysis of 16S rRNA gene in patients with ECOG-PS ≥3 (**a**) included significantly more oral streptococci than those in patients with ECOG-PS ≤2 (**b**) (*P* < 0.001). All members of the *Streptococcus mutans* and *S. mitis* groups, the *S. salivarius* group, and the *S. anginosus* group were included as “oral streptococci” except for *S. pneumoniae*. Abbreviation: ECOG-PS; european cooperative oncology group performance status

As the most detected bacterial phylotypes, oral streptococci were significantly more frequently detected in pneumonia patients with an ECOG-PS of ≥3 than those ≤2 (*P* < 0.001), while anaerobes were less frequently detected but not significantly different (Fig. 5). The percentages of oral streptococci were significantly highly detected in each BALF in patients with ECOG-PS ≥3 (Fig. 4e) than those with ECOG-PS ≤2 (Fig. 4f) (Fig. 3c, *P* < 0.001), and those of anaerobes were highly observed in each BALF in patients with ECOG-PS ≤2 (Fig. 4h) than ECOG-PS ≥3 (Fig. 4g), but not significantly different (Fig. 3d, *P* = 0.085).

## Discussion

This study firstly analyzed the relationship between the bacterial flora in the lung and the aspiration risks in patients with CAP and HCAP, and the bacterial floral analysis of 16S ribosomal RNA gene using the BALF specimens obtained from 83 CAP and 94 HCAP patients revealed that oral streptococci were the most detected bacterial phylotypes (Fig. 2c). This is in line with our previous results that demonstrated the significantly higher detection of oral streptococci in CAP patients [5]. However, the clinical implication of the detection of oral streptococci in relation to risk factors for aspiration and clinical background factors have not been fully understood. In this study, oral streptococci were significantly more frequently detected with high occupancy in pneumonia patients with aspiration risks than in those without aspiration risks (Figs. 3a and 4a, b). Moreover, the high occupancy of oral streptococci in each sample was strongly correlated with ECOG-PS ≥ 3 (Figs. 3c and 4e, f). In addition, this study firstly showed that having a poor ECOG-PS (≥3) and a history of pneumonia within the previous one year showed a greater correlation with the detection of oral streptococci in the BALF than having aspiration risks (Table 4).

Oral streptococci have been reported to be common causative pathogens of pulmonary abscesses and empyema thoracis [14–17], and the rate of oral streptococci as causative pathogens in patients with CAP or HCAP was reported to be 0.3–14.1 % [18–27]. Higher incidence of oral streptococci (22.3 %) as the most detected phylotypes in the BALF using the bacterial floral analysis of 16S rRNA gene was observed compared to the previous reports including our report of CAP patients (9.4 %) [5]. In addition, higher rates of oral streptococci were detected in both patients with aspiration risks (31.0 %) and without (14.7 %) (Fig. 3c, f) than the previously reported detection rates of oral streptococci in patients with aspiration (1.9–41.2 %) [14, 28–32] and non-aspiration pneumonia (3.6–9.1 %) [30, 32]. The different bacteriological detection methods may have influenced these different detection rates of oral streptococci [30, 32]. Conventional cultivation methods cannot evaluate the bacterial ratio in each sample, whereas the bacterial floral analysis of 16S rRNA gene we used can estimate the ratio of bacterial phylotypes in each sample. According to the result, the percentages of oral streptococci in each BALF sample in patients with aspiration risks were significantly higher than those in patients without aspiration risks (Fig. 3a).

The factors correlated with the detection of oral streptococci in pneumonia patients by simple regression analysis were similar to the previous reports (Table 3) [15]. Detection of oral streptococci by the bacterial floral analysis of 16S rRNA gene were strongly correlated to a risk of aspiration in this study, and the strongest correlation factor for the detection of oral streptococci in pneumonia patients in this study was a poor ECOG-PS (≥3) (Table 4).

Oral streptococci (22.3 %) were the most frequently detected bacterial phylotypes (Fig. 2c), whereas the conventional cultivation method demonstrated that oral streptococci and anaerobes were isolated in only 5.0 and 4.4 % of the same samples, respectively (Fig. 2b). In real-word clinical settings, oral streptococci and anaerobes might be considered as oral contaminants and could be underestimated by cultivation methods. We have previously shown that the bacterial floral analysis of 16S rRNA gene using BALF samples did not make remarkable oral floral contaminations in noninfectious patients with idiopathic interstitial pneumonias [5], and also all patients showed a total bacterial cell count of >10^4^ cells/mL in BALF that was a useful diagnostic criterion for pulmonary bacterial infection [5], therefore, we think that the oral streptococci and anaerobes that were highly detected in the lower respiratory tract in this study were reliable, and speculate that oral streptococci have important roles in patients with pneumonia with aspiration risk factors.

The detection rates of anaerobes in patients with aspiration pneumonia were 87.5–100 % in the studies using percutaneous transtracheal aspirates [28–30]. However, Marik et al. suggested that these high rates might be excessive [12], primarily due to their later sampling time of percutaneous transtracheal aspirates in the clinical course of the illness. This might include other complications such as abscesses, necrotizing pneumonia, or empyema in the previous studies [28–30], and transtracheal sampling may also have possibly provoked the aspiration of the oropharyngeal flora during the procedure. Anaerobes were detected in only 11.6 % of samples in other report evaluating using protected BALF specimens in patients with severe aspiration pneumonia in a population of institutionalized elderly [14], and no anaerobes were detected using a protected specimen brush [31, 33]. The bacterial floral analysis of 16S rRNA gene demonstrated that anaerobes were detected in 12.3 % of this study population (Fig. 2c), and significantly highly detected in patients without aspiration risks (17.9 %, Fig. 2i) than with aspiration risks (6.0 %, Fig. 2f). The percentages of anaerobes in each BALF sample in patients without aspiration risks were also significantly higher than those with aspiration risks (Figs. 3b and 4c, d), and according to these results, anaerobes might not be the primary cause of pneumonia in patients with aspiration risks. El-Solh et al. [14] also reported the relationship between the detection of anaerobes in the lower respiratory tract and oral hygiene, further studies are necessary to clarify these clinical relationships.

This study is associated with several limitations. First, this study was retrospective and subject to a recall bias. Second, the universal primers that were used in the bacterial floral analysis could not amplify all of the bacterial 16S rRNA genes, and the sensitivity of the primers was approximately 92 % of the total bacterial species registered in the Ribosomal Database Project II database, whereas no reported human causative pathogens were included in the remaining 8 %. Third, only approximately 100 clones were analyzed per library, suggesting that bacterial 16S rRNA gene sequences present at very small fractions (<1 % of each sample) in the sample may occasionally be undetectable. In addition, the sequencing depth was insufficient for the detection of mycobacterial species. Fourth, this study only included patients in whom bronchoscopic examination was performed.

## Conclusions

Using the bacterial floral analysis of 16S rRNA gene, oral streptococci were the most detected bacterial phylotypes in CAP and HCAP patients with aspiration risks, and both simple and multiple regression analyses demonstrated that the detection of oral streptococci in the BALF by the bacterial floral analysis of 16S rRNA gene was strongly related to poor ECOG-PS (≥3). The evaluation of the patients’ physical activities to estimate the aspiration risks and bacterial etiologies might be important, and a poor ECOG-PS might be a clue to the higher detection rate of oral streptococci in the lower respiratory tract in patients with CAP and HCAP.

### Ethics approval and consent to participate

This study was approved by the Human and Animal Ethics Review Committee of the University of Occupational and Environmental Health, Japan (No.09–118), and all patients provided their written informed consent.

### Availability of data and materials

We declare that the data supporting the conclusions of this article are fully described in the article.

## Abbreviations

BALF: bronchoalveolar lavage fluid
BLAST: basic local alignment search tool
BMI: body mass index
CAP: community-acquired pneumonia
CT: computed tomography
ECOG-PS: eastern cooperative oncology group-performance status
HCAP: healthcare-associated pneumonia
PCR: polymerase chain reaction
PSI: pneumonia severity index
VAP: ventilator-associated pneumonia
